# Supplementary material for: PmeR, a TetR-like transcriptional regulator, is involved in both auxin signaling and virulence in the plant pathogen Pseudomonas syringae strain PtoDC3000
Source: mBio. 2025 Aug 20;16(9):e01152-25. doi: 10.1128/mbio.01152-25 (PMC12421961; doi:10.1128/mbio.01152-25)
Supplement: Supplemental Figures and Tables — Figures S1 to S6; Tables S1 and S2. [file mbio.01152-25-s0001.pdf]

Supplemental Materials

PmeR, a TetR-like transcriptional regulator, is involved in both auxin signaling and virulence in the plant pathogen *Pseudomonas syringae* strain *Pto*DC3000.

Chia-Yun Lee, Maya Irvine, Barbara Kunkel*

Department of Biology, Washington University in St. Louis, St. Louis, Missouri, United States of America

* Corresponding author

E-mail: kunkel@wustl.edu

The PDF file includes:

Figures S1- S6

Tables S1- S2

Supplemental Figures

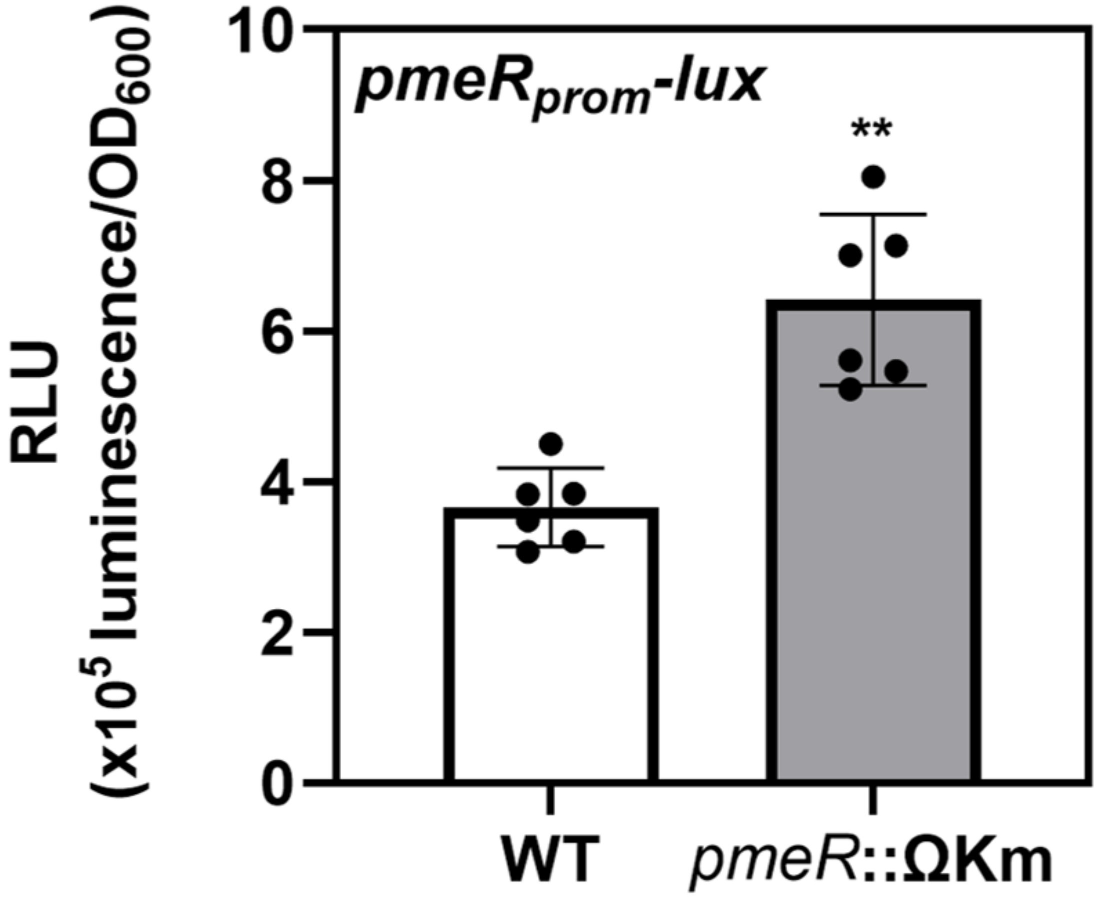

Fig S1. *pmeR* represses its own expression. Luciferase activity of the *pmeR$_{prom}$-lux* reporter in wild-type *Pto*DC3000 (WT, white bar) and the *pmeR*::ΩKm mutant (grey bar) in Hrp de-repressing medium (HDM). Data represents mean luminescence normalized to cell density (OD_{600}), plotted as relative luminescence units (RLU) ± standard deviation (SD), and are compiled from two independent experiments (n = 6). Asterisks indicate significant differences between WT and *pmeR*::ΩKm mutant strains as determined by Student's *t*-test (**: $p < 0.01$).

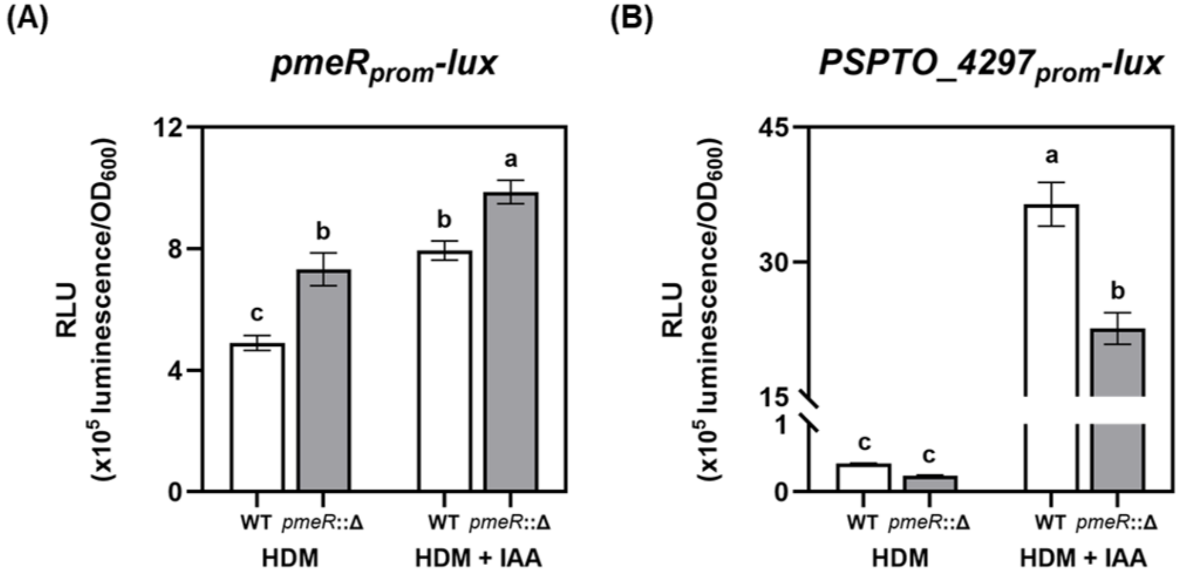

Fig S2. An independent deletion mutant of *pmeR* exhibits elevated expression of *pmeR* and reduced IAA induction of *PSPTO_4297*. Expression of (A) *pmeR$_{prom}$-lux* and (B) *PSPTO_4297$_{prom}$-lux* in wild-type *Pto*DC3000 (WT, white bar) and the *pmeR* deletion mutant (*pmeR*::Δ, grey bar) in response to IAA at 90 minutes post-treatment. Data are from a representative experiment (n = 6) and plotted as the mean of relative luminescence units (RLU) ± SD. Similar results were obtained in two additional independent experiments. Letters indicate significant differences between treatments as determined by ANOVA followed by Tukey's HSD test ($p < 0.05$). HDM: Hrp de-repressing medium; HDM + IAA: HDM supplemented with 100 µM IAA.

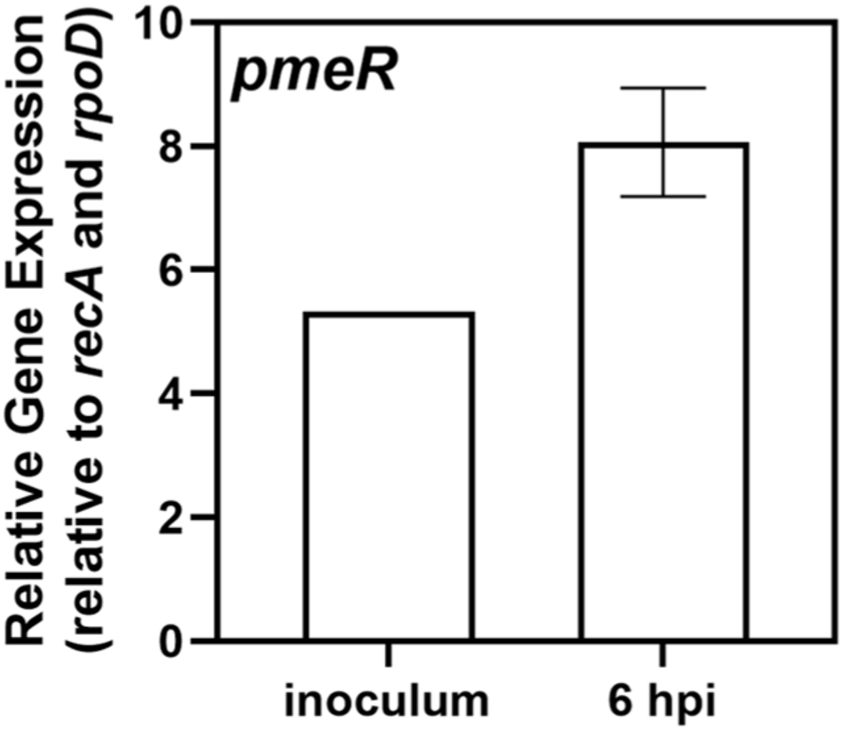

Fig S3. *pmeR* is expressed *in planta*. Expression of *pmeR* in *Pto*DC3000 growing in *A. thaliana* Col-0 plants. To quantify bacterial gene expression, infected leaves were harvested 6 hours after inoculation, and total RNA was isolated and used for qRT-PCR. Bacterial RNA from 1 mL of the initial *Pto*DC3000 cell resuspension was isolated and served as a control (inoculum, n = 1). The relative expression was calculated by normalizing *pmeR* expression to the expression of two reference genes, *rpoD* and *recA.* Data are from a representative experiment (n = 3) and plotted as the mean of relative gene expression ± SD. Similar results were obtained in a second independent experiment.

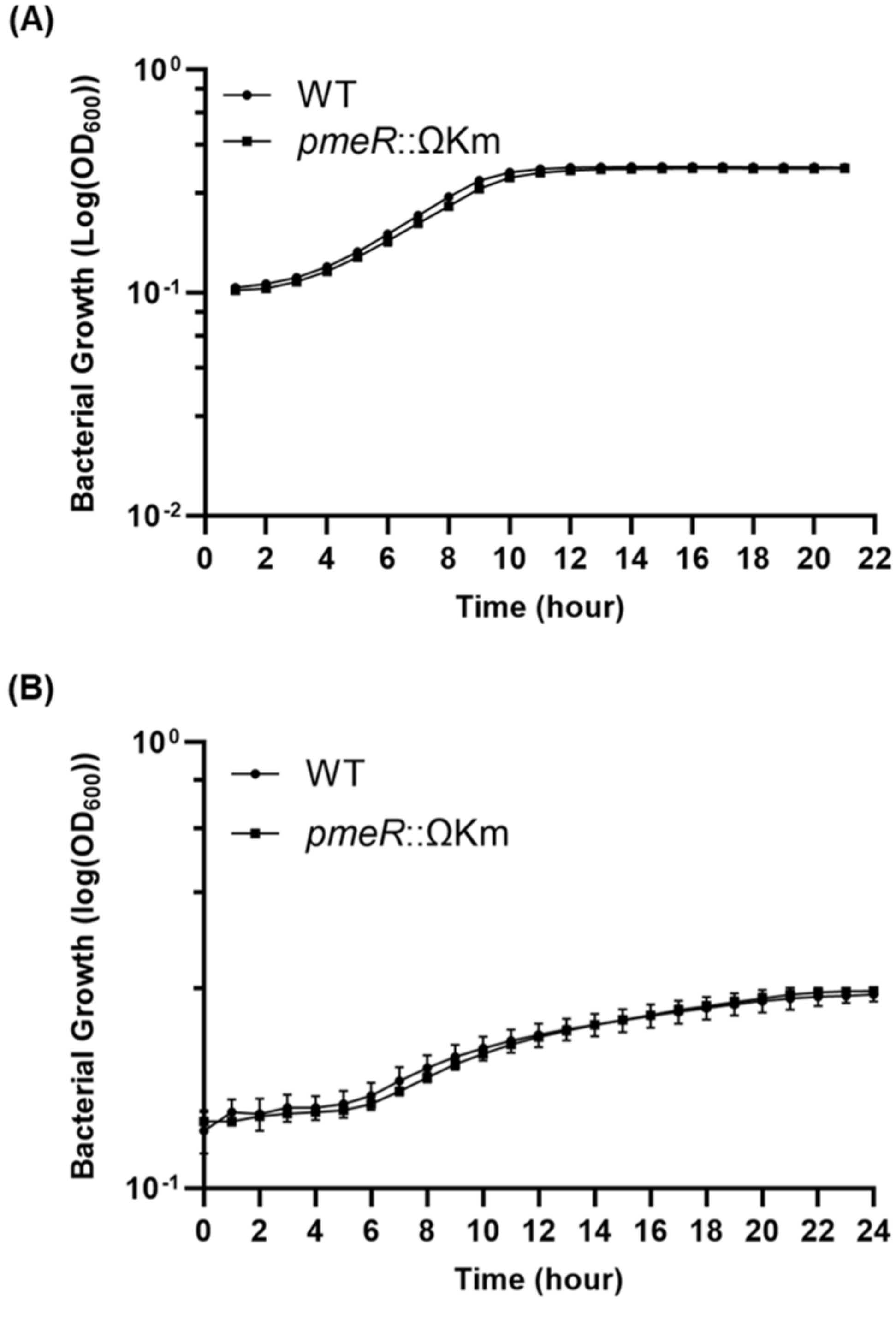

Fig S4. Growth of the *pmeR*::ΩKan mutant in culture. Bacterial growth of WT and *pmeR*::ΩKm in (A) rich (NYG pH 5.7) and (B) minimal media (HDM). Data are from one representative experiment and shown as mean ± SD (n = 2). No significant difference between strains was observed as determined by Student's *t*-test. Similar results were obtained in an additional independent experiment.

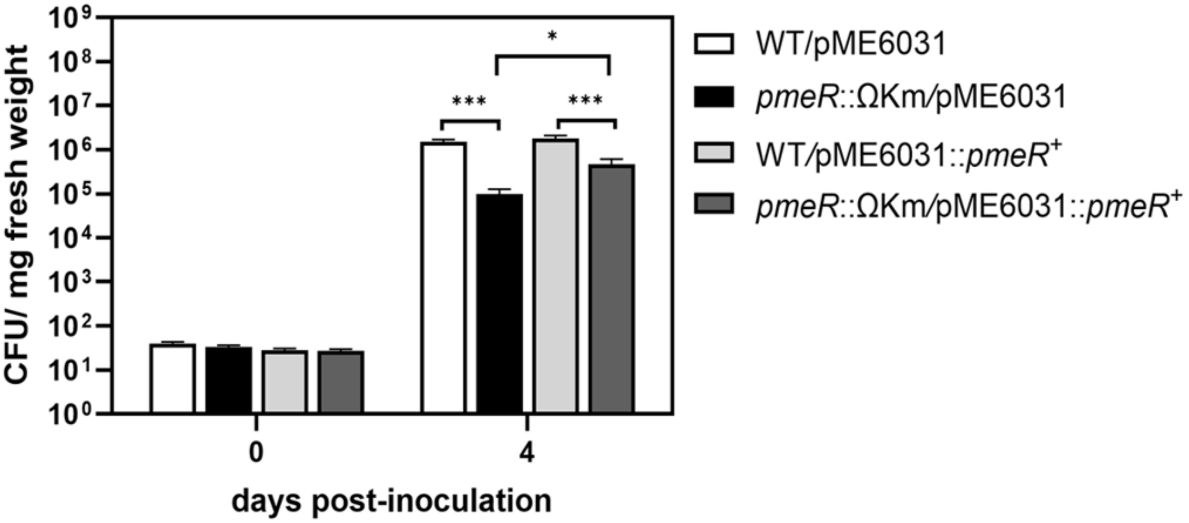

Fig S5. The wild-type *pmeR* gene partially complements the growth defect of *Pto*DC3000 in *A. thaliana*. Four-week-old wild-type *A. thaliana* Col-0 were infiltrated with ~ 1 x 10^5 CFU/mL of bacteria. Bacterial growth in infiltrated leaves was quantified at 0 and 4 dpi. Data from two representative experiments are combined and shown as mean ± standard error (SE) (n = 8 for 0 dpi, n = 12 for 4 dpi). Asterisk indicates significant differences between treatments as determined by Student's *t*-test (*: $p < 0.05$; ***: $p < 0.001$). CFU: Colony forming units.

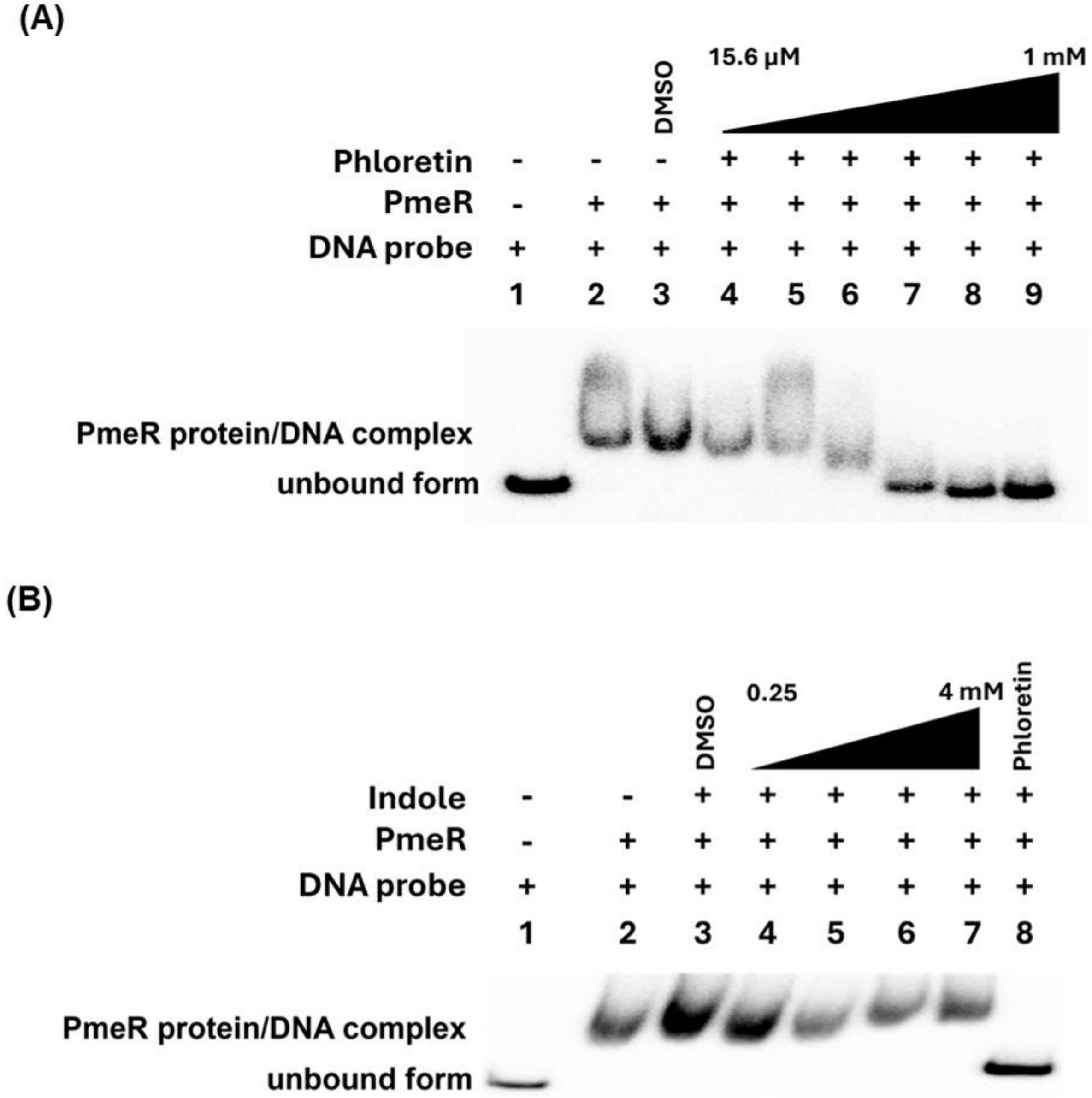

Fig S6. Phloretin disrupts the PmeR/DNA interaction. (A) The phosphor image of electrophoresis mobility shift assays (EMSA) confirms that phloretin disrupts the PmeR/DNA interaction. Increasing concentrations of phloretin (15.6 µM to 1 mM) were added to the co-incubation of 1.5 nM DNA probe and 6.25 nM purified PmeR protein. (B) The phosphor image of EMSA demonstrates that indole does not disrupt the PmeR/DNA interaction. Increasing concentrations of indole (0.25 mM to 4 mM) were added to the co-incubation of 1.5 nM DNA probe and 6.25 nM purified PmeR protein. For data shown in panels A and B, similar results were seen in two additional independent experiments.

Supplemental Tables

Table S1. Bacterial strains and plasmids used in this study.

Strain/Plasmid	Characteristics [a]	Reference
***P. syringae* strains**		
*Pto*DC3000 (WT)	Derivative of NCPPB1106; Rif^R	(34)
*Pto*DC3000-MG (WT)	WT parent of *mexA*::ΩKan and *pmeR*::ΩKan mutants; Rif^R	(12)
mexA::ΩKm	Rif^R, Kan^R	(12)
pmeR::ΩKm	Rif^R, Kan^R	(12)
*Pto*DC3000/pME6031::*lux*	Rif^R, Tet^R	(15)
*Pto*DC3000/pME6031::*pmeR$_{prom}$-lux*	Rif^R, Tet^R	This study
pmeR::ΩKm/pME6031::*pmeR$_{prom}$-lux*	Rif^R, Kan^R, Tet^R	This study
*Pto*DC3000/pME6031::*PSPTO_4297$_{prom}$-lux*	Rif^R, Tet^R	This study
pmeR::ΩKm/pME6031::*PSPTO_4297$_{prom}$-lux*	Rif^R, Kan^R, Tet^R	This study
*Pto*DC3000::pK18*pmeR*Δ	*Pto*DC3000 with pK18*pmeR*Δ integrated at *pmeR* locus; Rif^R, Kan^R	This study
pmeR::Δ	*pmeR* in-frame deletion mutant; Rif^R	This study
pmeR::Δ/pME6031::*pmeR$_{prom}$-lux*	Rif^R, Tet^R	This study
***E.coli* strains**		
DH5α	*recA, lacZΔM15*	Bethesda Research Lab
MM294A/pRK2013-Cm	Triparental mating helper strain carrying pRK2013; Cm^R	(40)
BL21 (DE3)/pET-28a-PmeR	Kan^R	This study
**Plasmids**		
pRK2013	Helper plasmid for triparental mating; Cm^R	(40)
pME6031	Broad host range plasmid, stable in *P. syringae*; Tet^R	(14)
pME6031::*lux*	Vector for generating *promoter-lux* fusions; Tet^R	(15)
pME6031::*pmeR $_{prom}$-lux*	~250 bp of *pmeR* (*PSPTO_4302*) regulatory region in pME6031::*lux*; Tet^R	This study
pME6031::*PSPTO_4297$_{prom}$-lux*	417 bp of *PSPTO_4297* regulatory region in pME6031::*lux*; Tet^R	(15)
pME6031::*pmeR*$^+$	936-bp genomic clone of wild-type *pmeR* (*PSPTO_4302)* on pME6031; Tet^R	This study
pK18mobsacB	Plasmid for gene replacement using sucrose counterselection; Kan^R	(42)
pK18*pmeR*Δ	*pmeR*::Δ construct on pK18mobSacB; Kan^R	This study
pET-28a-(+)	Kan^R	Novagen
pET-28a-PmeR	His-tagged PmeR expression construct; Kan^R	This study

[a] Rif^R, Kan^R, Tet^R, Cm^R indicate resistance to rifampicin, kanamycin, tetracycline, and chloramphenicol, respectively.

Table S2. Primers used in this study.

Primer Name	Primer Sequence	Reference
**Construction of $\boldsymbol{pmeR_{prom}}$-*lux* reporter fusion, amplification of EMSA probe, and complementation**		
FPlux KpnI-pmeR	GGGGTACCGAAGATCCTCGGGGCAGG	This study.
RPlux EcoRI-pmeR	CGGAATTCGGTGCGACGAACCATGG	This study.
**Construction of *pmeR* deletion plasmid**		
pmeRupstreamFWD[a]	GGTACCGGATCCCTGGTCAACCGACACTTC	This study.
pmeRupstreamREV[a,b]	GCG***CTATAACCTTTC*GCGACGAACCAT**GGCAATAAG	This study.
pmeRdownstreamFWD[a,b]	GCC**ATGGTTCGTCGC*GAAAGGTTATAG***CGCCTG	This study.
pmeRdownstreamREV[a]	CAGGTCTCTAGACGGTAAATACTGGTGACC	This study.
pK18vectorFWD[a]	CACCAGTATTTACCGTCTAGAGTCGACCTGCAG	This study.
pK18vectorREV[a]	GTGTCGGTTGACCAGGGATCCCCGGGTACCGAG	This study.
**qRT-PCR**		
PSPTO_0371 (*iaaL*) F	GTCTGGGCATCATTCAAC	(10)
PSPTO_0371 (*iaaL*) R	ACGGTCACCAGTTCTTT	(10)
PSPTO_0537 (*rpoD*) F	GAAGTTGACGAAAGCTGGACCG	(9)
PSPTO_0537 (*rpoD*) R	CGACGGTTGATGTCCTTGATCTC	(9)
PSPTO_1404 (*hrpL*) F	TCAGGAAAGCTGGGAAGACGAAGT	(37)
PSPTO_1404 (*hrpL*)R	ATGTTCGACGGCAGGCAATCAATG	(37)
PSPTO_1824 F	GTTGAGCAGCCACTTTG	(10)
PSPTO_1824 R	AGCTTTATTAGTGGCATTGG	(10)
PSPTO_3549 (*aefR*) F	GCCTCACCGATCAGAAA	(10)
PSPTO_3549 (*aefR*) R	GTCCATGCTGGTGATCT	(10)
PSPTO_3576 (*tvrR*) F	GGCTCGCAACGGCCCATCTG	(9)
PSPTO_3576 (*tvrR*) R	CATGCGGTAGACGGCCAGCG	(9)
PSPTO_4033 (*recA*) F	TAGAACTTCAGCGCGTTACC	(43)
PSPTO_4033 (*recA*) R	GCCAACTGCCTGGTTATCT	(43)
PSPTO_4302 (*pmeR*) F	GATGCTCGACAGCCTTC	This study.
PSPTO_4302 (*pmeR*)R	TCAACAGGTTCCTCATGC	This study.
PSPTO_4303 (*mexA*) F	GTCAACGGCATCATTCTC	(10)
PSPTO_4303 (*mexA*) R	GCGTTGGCTTCATAGATAG	(10)
PSPTO_4297 F	CTTTCTCTGTTCTGGCTTC	(10)
PSPTO_4297 R	GGGAGGCTTTGGACATA	(10)

[a]Sequences homologous to *Pto*DC3000 genome are underlined

[b]Sequences corresponding to the first 4 codons of *pmeR* are in bold, the last 4 codons are in bold italics.
